# The role of informal caregivers in suicide prevention for men– findings from a psychological autopsy study

**DOI:** 10.1186/s12889-025-21594-x

**Published:** 2025-01-29

**Authors:** Laura Hofmann, Anna-Lena Springer, Birgit Wagner

**Affiliations:** Department of Clinical Psychology, Medical School Berlin, Ruedesheimer Straße 50, 14197 Berlin, Germany

**Keywords:** Suicide prevention, Mental health, Gender, Psychological autopsy, Caregiver burden

## Abstract

**Background:**

Men have a significantly higher risk of dying by suicide than women and at the same time are less likely to make use of psychosocial support services. Therefore, informal caregivers who care for a person at risk play a significant role. This study aims to highlight the distress and support needs of informal caregivers for men in suicidal crises.

**Methods:**

*N* = 15 participants who lost a man to suicide in the last 3–12 months were interviewed using psychological autopsy interviews. The interviews were analyzed using a comprehensive category system while following a deductive-inductive approach.

**Results:**

The majority of participants reported experiencing significant stress as well as anxiety about leaving the affected men unsupervised. Only four participants indicated that they openly discussed their stress with their social environment, and just two sought professional support. Notably, only two caregivers anticipated the possibility of suicide. There was also little professional support for caregivers, resulting in participants using internet resources to inform themselves about suicidal ideation and behavior.

**Conclusion:**

These findings highlight the significant stress and burden experienced by informal caregivers in suicide prevention for men. Lack of open communication and insufficient access to support exacerbate the emotional burden on caregivers. These results highlight the need for accessible resources and assistance to better support both caregivers and the individuals they care for.

**Trial registration:**

German Clinical Trials Register, DRKS00030758, Registered on 11.11.2022.

## Introduction

Caring for a loved one with suicidal ideation and behavior involves not only addressing the needs of the affected individual but also navigating considerable challenges that can deeply affect the caregiver’s well-being and quality of life. Informal caregivers are individuals who provide ongoing support and assistance to a person with a physical illness, disability, or mental health disorder [1, 2]. Informal care includes any support provided by a person from the immediate environment while informal caregivers are not professionally qualified or paid [3]. This role is usually filled by close family members or partners with women being significantly more likely to be caregivers than men [2]. There are now numerous studies that examine the burden on caregivers in the care of people with mental health disorders and also specifically with suicidal ideation and behavior [4–7].

The distress experienced by informal caregivers is divided into objective and subjective burden [8]. Objective burden refers to measurable and direct effects, such as social isolation, financial difficulties, challenges in managing household responsibilities, and restrictions in the caregiver’s daily life [1, 9]. In contrast, subjective burden encompasses psychosocial and physical issues, including feelings of shame, grief, stigmatization, worry, depressive symptoms, and physical complaints [1, 10]. Informal caregivers are confronted with the dual challenge of caring for the affected individual while also maintaining their own daily lives [9]. This challenge is especially evident in cases of suicidality, where caregivers often remain on constant alert, hesitant to leave the individual alone due to fear, and hyper-vigilant in monitoring for potential warning signs. Fogarty and colleagues [7] found in their analysis of interviews with men who have attempted suicide and their informal caregivers the significant challenges caregivers face in suicide prevention. While caregivers aim to monitor and support the individual closely, this can sometimes be perceived by the person at risk as invasive or irritating, leading to a tension between respecting the individual’s autonomy and the need to intervene. Additionally, informal caregivers often struggle with a lack of knowledge in recognizing warning signs and risk factors, which can result in inadequate intervention and uncertainty in how to appropriately support those at risk [11, 12].

The burden of informal caregivers is further compounded by the significant time commitment of informal caregivers, who often try to balance their caregiving responsibilities. A study by Hastrup et al. [13] found that informal caregivers for individuals with mental health disorders dedicate an average of 60 h per week to caregiving. Additionally, informal caregivers for mental health disorders are more often parents or partners. These caregivers also report a lower quality of life and higher levels of subjective burden [13]. This heightened stress may be attributed to the feelings of helplessness that mental health disorders can evoke, often due to a lack of knowledge about the disorder and limited involvement of family members in the treatment process [1]. Additionally, factors such as being female, younger age, lower socio-economic status, and living with the affected individual are associated with increased stress levels [10, 13, 14].

It is evident that relatives play a crucial role as informal caregivers for individuals with mental health disorders, particularly in suicide prevention [7]. In this context, close family members often serve not only as caregivers but also as gatekeepers. As caregivers, they provide essential support to the affected individual, and as gatekeepers, they are able to recognize early warning signs of suicide risk and facilitate timely access to support services [15]. Due to gatekeepers being in personal contact with individuals at risk, they are more likely to notice changes in behavior and intervene by engaging in conversations and connecting individuals to support services [12]. Gatekeepers are individuals close to a person at risk who, due to their proximity, are better equipped to identify warning signs and connect the person to appropriate help [16]. A prevalent problem is the often insufficient knowledge regarding warning signs, symptoms and available support, alongside the dual responsibility of informal caregivers who also serve as gatekeepers [17, 18]. Several studies have highlighted that the effectiveness of gatekeepers is significantly associated with their level of knowledge about suicidal ideation and behavior and appropriate intervention strategies [19, 20]. Insecurities in managing individuals at risk, combined with misconceptions about suicidal ideation and behavior, often leads to reduced intervention behavior.

Gatekeepers play a particularly important role in the prevention of suicide among men. The increased rate of suicide among men is a global phenomenon. Men are about 3–4 times more likely to die by suicide than women, who more often attempt suicide [21]. The so-called gender paradox highlights the need for a better understanding of gender-specific risk factors and tailored suicide prevention interventions for men [22]. Men often show different symptoms in the context of mental health disorders than is generally known [23]. In the context of depressive episodes, for example, men show symptoms such as aggressiveness, risky behavior, alcohol and drug abuse and irritability [23, 24]. As a result, the suicide risk of men is often not recognized as these symptoms are often misinterpreted [25]. Men do communicate suicidal thoughts but these are often not interpreted correctly by their surroundings and taken as warning signals [18]. Men are also less likely to use the healthcare system and experience psychosocial support services as less helpful [26]. A study by Spahn et al. [27] demonstrated that, unlike women, men are more inclined to seek support from their social environment rather than from professional services during a crisis, underscoring the importance of family and friends. Despite this, the specific role of relatives as informal caregivers in suicide prevention among men remains poorly understood. Notably, male gender and younger age of the person being cared for, are linked to heightened stress levels among caregivers [10, 28, 29]. Given both the significant burden on caregivers and their critical role in suicide prevention, this study aims to examine the role of caregivers in the suicide prevention of men.

This study is a secondary analysis of psychological autopsy interviews conducted with informal caregivers of men who have died by suicide. It explores the following questions: (a) What concerns do caregivers experience when interacting with suicidal men? (b) How do caregivers communicate their distress? (c) How do informal caregiver cope with their distress?, (d) What kind of support do caregivers receive?, and (e) What additional support services would be necessary?

## Methods

### Study design

The present study was conducted as part of the collaborative project MEN-ACCESS: Suicide Prevention for Men. The project consists of the University of Leipzig, the Medical School Berlin and the University of Bielefeld and is funded by the German Federal Center for Health Education (Project Identifier: Z2/95F12003B). The overall aim of the project is to develop two gender-specific e-learning tools for men at increased risk of suicide and for gatekeepers. In addition, it aims to identify specific risk factors, barriers, experiences with help services and the role of gatekeepers. The present study follows a mixed-methods approach. Participants first completed a quantitative online questionnaire, which collected demographic information about participants, as well as information about the suicide, mental health, and physical health of the deceased men. Subsequently, psychological autopsy interviews were conducted with the caregivers, which serve the retrospective analysis of suicides and are intended to provide information about the suicide analyzing the interview material, suicide notes, and medical reports [30].

The study has been approved by the Ethics Committee of the Medical School Berlin (registration number MSB-2021/67) on July 14th, 2021. Part of the data focusing on the behavior of men before a suicide has already been published [18]. The participants needed to provide written informed consent prior to participation.

### Data collection and sample

A total of *N* = 15 informal caregivers were included in the study. Data collection took place in July and August 2021 in Germany. Recruitment was primarily via social media (Instagram, Twitter, Facebook) as well as support groups and self-help groups for suicide bereaved individuals. The following inclusion criteria had to be met for participation: [1] loss of a close male relative (father, brother, son, partner) through suicide in the last 3–12 months [2], at least 18 years of age [3], sufficient German language skills, and [4] a signed informed consent form.

### Sample characteristics

#### Participants

A total of *N* = 15 female caregivers aged between 28 and 67 years with an average age of *M* = 42.73 (*SD* = 12.78) years participated in the study. The study sample consisted of individuals of Western European ethnic background and participants predominantly had a higher socioeconomic status. On average, the suicide happened 6.47 (SD = 2.39) months ago. Six participants (40.0%) lost their brother, four (26.7%) their son, three (20.0%) their partner and two (13.3%) lost their father. Overall, three (20.0%) of the participants found the deceased themselves, in four cases (26.7%) another family member found the deceased, in another four cases (26.7%) the deceased was found by the police or medical staff, and in four cases (26.7%) by strangers.

#### Deceased men

The deceased were aged between 17 and 75 years with an average age of 38.47 (*SD* = 14.85) at the time of the suicide. The majority was single (*n* = 8, 53.3%), six (40.0%) were married or in a relationship and one (6.7%) was divorced. The majority (*n* = 13) had at least one diagnosis of a mental health disorder and almost half (*n* = 7) took psychotropic medication. The most common used suicide method was hanging (*n* = 6), *n* = 4 had at least one suicide attempt in the past and *n* = 4 of the deceased left a suicide note. Overall, *n* = 10 of the deceased men had professional support in the past. Table 1 presents additional information on the suicide.

**Table 1:** Sociodemographic characteristics of deceased men (*N* = 15)

	M (SD)	Range
Age in years	38.47 (14.85)	17–75
	***n***	**%**
Method of suicide		
Hanging	6	40.0
Jumping	4	26.7
Intoxication	1	6.7
Rail suicide	1	6.7
Other	3	20.0
Diagnosis^1^	13	86.7
Depression	10	66.7
Schizophrenia	2	13.3
Substance Abuse	3	20.0
Anxiety Disorder	1	6.7
Farewell letter (yes)	4	26.7
Prior suicide attempts (yes)	4	26.7
In therapy at time of death	5	33.3
Therapy in the past	10	66.7
Psychotropic medication	7	46.7
Under influence at time of suicide	2	13.3

Note. ^1^Multiple answers possible

### Procedure

A total of *N* = 24 interested individuals contacted the study coordinator by e-mail, who then sent them information about the study and the consent form. Seven individuals had to be excluded in advance because the suicide had occurred more than twelve months ago. Two of the participants did not respond after sending the study information, so that a total of 15 participants signed the consent form and participated in the study. Participants were then emailed the link to an online questionnaire, which took about 15 min to complete. At the end of the questionnaire, participants had the opportunity to indicate possible dates for the interviews. Once they completed the questionnaire, the study coordinator sent potential dates with a request for confirmation. The semi-structured interviews were conducted via telephone by a psychological psychotherapist. Interviews were recorded using a voice recorder without an Internet connection and lasted an average of 77.40 (*SD* = 23.38) minutes.

At the beginning of the interview, the interviewer briefly introduced herself and gave an overview of the interview process. Subsequently, questions on behalf of the participants could be addressed. Participants were also informed that breaks were possible at all times and that they could discontinue their participation in the study at any time. Since additional information such as suicide notes or medical reports are also relevant in psychological autopsy interviews, participants were asked to read these aloud. After the interview, participants were asked how they were feeling and, if necessary, if they would like to talk further about their stress.

The quantitative data was collected pseudonymized using an individual code. A coding list existed in paper form, which linked the code with the name of the participant. This list was kept in a data protection cabinet and was only accessible to project staff and was shredded following data collection, so it was not possible to subsequently link the dataset to an individual. The interviews were also saved under the respective pseudonym. During transcription, all personal data was anonymized so that no identification of the participant or deceased person is possible. The transcription was based on the content-semantic approach by Kuckartz [31]. The language was smoothed slightly, stuttering, and in-between sounds were removed, and dialectal words were rendered in standard language. This increases the readability of the transcript, since in this case the content is more important than the way of speaking.

### Measures

#### Quantitative measures

The quantitative online questionnaire collected the following information: demographic data of the participant and the deceased person, information on the suicide, and symptoms of depressiveness and anxiety in the deceased person. Since this publication focuses on informal caregivers, the questionnaires on the mental health of the deceased are not included here.

#### Demographic variables

The following socio-demographic data of the participants and the deceased were collected: Age, gender, marital status, living situation, level of education, and degree of kinship.

#### Information on the suicide and the mental health

We assessed the method of the suicide, whether there was a suicide note, if there were any suicide attempts in the past, and whether the deceased individual was under the influence of alcohol and/or drugs at the time of the suicide.

#### Mental health of the deceased

Participants were asked to state the diagnoses of the deceased person, if available, and whether the person was in therapy at the time of the suicide or in the past. In addition, it was asked if the person was taking psychotropic medication.

#### The interview guide

In total, the interview was developed following existing autopsy studies and guidelines [32–34] with a focus on male suicide and their informal caregivers. Psychological autopsy interviews are a common method in suicide research, in which relatives and the social surroundings, but also doctors or other contact persons of people who have died by suicide are interviewed. In addition, doctor’s letters, farewell letters or other documents are analyzed, if available. The aim is to shed light on the time before the suicide to better understand the suicide. The interview guide consisted of a total of six main categories: [1] behavior in the days before the suicide and information about the suicide [2], information about the mental health of the deceased and support services [3], information about the physical health of the deceased [4], information on social contacts and family [5], information on the professional and financial situation of the deceased, and [6] distress and support for informal caregivers. If available, caregivers were asked to read doctor’s notes and farewell letters aloud during the interview.

### Qualitative and statistical analyses

Quantitative data were processed and analyzed using SPSS Version 28 [35] while presenting frequencies for categorical variables and means and standard deviations for continuous variables.

The analysis of the qualitative data followed a deductive-inductive approach according to Mayring [36]. The approach includes the following steps: [1] preparation and organization of the data [2], dividing the data into smaller datasets [3], creating codes while reading the first dataset [4], rereading the first dataset and applying the codes [5], reading the next dataset while applying the codes [6], creating new codes when the existing codes do not fit [7], recoding all datasets once again while applying the new codes [8], repeating all the steps until all datasets are encoded. A category system always consists of main categories, which are developed deductively before the analysis based on theoretical considerations. A main category then usually contains several associated subcategories, which form the inductive category system and are developed during the analysis of the material. The analysis was carried out with the MAXQDA Software [37] by two individual raters.

## Results

### Qualitative findings

The category from the interview guide focusing on the role of informal caregivers is comprised of a total of five subcategories: [1] own concerns and coping with concerns [2], communication of own distress [3], coping with the male relative’s psychological distress prior to the suicide [4], support received before suicide, and [5] ideas and needs regarding future support services for informal caregivers. Doctor’s notes and farewell letters were not analyzed for this study.

#### Own concerns and coping

This category encompasses caregivers’ general concerns about the individual, their specific worries regarding suicidal thoughts, and the strategies they employed to address these concerns.

Overall, about half (*n* = 7) of the participants reported that they were generally worried about their male relative, but not all were necessarily worried that he might have suicidal thoughts. In most cases, participants were concerned about the deceased prior to the suicide due to deteriorating mental health or because they observed changes in the person.

*The therapy was ending and he suddenly got the idea that his medication was no longer working and he questioned everything (…) I was quite worried about all that. (Wife*,* 30y).*

Two mothers reported that they had always been worried about their son in general. In these cases, the sons experienced periods of distress, and the mothers consistently expressed concern.

*I was always worried about him*,* since he was young*,* because he thinks too much (…) and that he is struggling with new things. (Mother*,* 45y)*

However, two participants reported that they were worried due to potential suicidal tendencies. Both, however, were already aware that their male relative was having suicidal thoughts and, because of this, were increasingly worried that something might happen. The other caregivers reported that, despite the individual’s severe condition, they did not perceive suicide as a plausible or imminent risk.

*I had this thought from time to time (…) I always texted him*,* sleep well. On WhatsApp. And I thought*,* Gosh*,* what happens*,* when I do not text him? (Mother*,* 67y)*

Three participants reported that they did not feel concerned about their male relative, as he was attending therapy and they believed that his condition would improve with professional support.

*And then I said to my boyfriend: We do not have to worry that much*,* he is getting help now and it is going to be okay. The first step is realizing that he needs help. (Sister*,* 28y)*

Two participants stated that they did not feel concerned because the deceased repeatedly assured that everything was fine and that nothing will happen.

*I trusted him. Because I thought*,* okay*,* if he says he is fine*,* then it will be fine. (Daughter*,* 29y)*

#### Communication of own distress

This category includes whether participants could communicate their distress and concerns with their male relative or with their social environment and what their experiences were. Only 4 of the caregivers reported that they talked to the deceased person about their fears or turned to their surroundings. In all four cases, however, the participants reported that they felt that addressing their concerns had no impact, at least on the affected individual.

*And I often saw him sitting there apathetically and I said: I somehow have the feeling that you do not notice anything anymore*,* Dad (…) And he just said*,* no*,* now leave me alone*,* you are annoying. (Daughter*,* 45y)*

Participants reported either, as in the example above, that the person reacted with annoyance and became defensive or downplayed the stress to reassure those around them.

*I said*,* hey*,* you are overworked. And he said*,* oh*,* it is not that bad. And he denied everything (…) So that was actually more of a short conversation. (Mother*,* 61y)*

Participants also reported feeling generally apprehensive about initiating such conversations. If the affected individual minimized their stress, participants often felt a sense of relief, even though they were aware that the person was significantly burdened.

Participants who did not express their concerns to the affected male reported insecurity about how to approach the conversation. They also reported that they had never talked to the deceased about his feelings or stress before and that he was not the type to talk about it.

*I already saw that he is not doing well. He had tears in his eyes. But I knew him*,* and I knew I could not just say*,* ‘now tell me*,* what is going on’. Because he was never the type to talk about that. (Sister*,* 28y)*

Only a few participants reported that they explicitly talked about their own burden with other family members perceiving it as helpful. Although the concern for the male relative was present in the family, many stated that their own stress tended to recede into the background. The focus was on the concern for the person at risk.

*During the time when he was still alive (…) I did not talk about it with anyone. It was not really a topic in the family either. (Sister*,* 49y)*

#### Coping with the male relative’s psychological distress prior to the suicide

This category covers how informal caregivers dealt with the mental distress or even mental health disorder of the male relative before they died through suicide. They were also asked whether they were involved in their relative’s therapy and how they interacted with the person in everyday life. Seven of the participants stated that they had dealt very openly with the man’s mental condition, had tried to listen to him and offered to talk to him. They reported attempting to approach the individual with sensitivity and understanding while avoiding excessive leniency or overindulgence.

*We did not pamper him too much*,* but we did not dismiss it either. If you somehow make too much fuss about it*,* then I think the person feels confirmed in all his fears. But if you dismiss it and say*,* you are out of your mind*,* then it would also be fatal. So I think we have all found a good mix. (Daughter*,* 45y)*

It was also considered especially helpful that caregivers informed themselves about mental health disorders, mostly by reading books, on the internet or by watching documentaries.

*So there is online support like a family coach for depression and stuff like that*,* we did that every now and then and we read books once in a while. (Sister*,* 34y)*

In seven cases, participants reported that they had made specific offers of support to their relative at risk. Two families set up an emergency number that the affected person could call at any time if he was feeling worse. In other cases, the participants supported the deceased in implementing a daily structure, took over small tasks in the household or assisted him with everyday things such as grocery shopping or cooking.

*We then set up a regular daily routine for him and tried to take some of the pressure off him. (Wife*,* 30y)*

Four participants also reported that they looked for support services together, made appointments, or even accompanying the person at risk to appointments.

*I had to deal with a lot of doctors. Because I just wanted to take him to the doctor. Because I realized something was not right. I was at my limit. I could not take it anymore. (Wife*,* 54y)*

However, hardly any caregivers were involved in the therapy. Three participants also reported highly negative experiences when they tried to talk to clinic staff about the acute suicidality of their male relative and the staff was not willing to listen to them.

*I said ‘I would like to talk to the doctor’. And then she said to my son ‘Your mother wants to talk to me about you’. [He said] no. I said ‘Well then do not talk to me*,* I just want to say something*,* I think that was a suicide attempt’. [She said] ‘I do not care. Your son does not want us to talk’. (Mother*,* 53y)*

Five participants reported that they no longer left the male at risk alone. In some cases, family members took turns to be with him all the time, or family members moved in with him or stayed overnight to watch over him. Some stated that they no longer dared to leave the house and canceled all appointments out of fear of leaving the affected person alone. Although many caregivers did not anticipate suicide, a significant number reported feeling the need to provide constant supervision for the individual.

*And he was always very attached to me and I always spent all my free time with him (…) I did not feel like going out at all. Then the first lockdown came*,* and you were not allowed to leave the house. And I said*,* thank God. Thank God. I just do not have to make any excuses anymore. (Mother*,* 53y)*

However, in two cases, participants also reported being offended or even angry at times. One participant reported feeling angry and scolding her partner when he expressed suicidal. Another participant shared that she sometimes felt offended when her brother rejected her help, despite the significant effort she invested.

*But in the meantime*,* I was also a bit offended because I thought*,* ‘Gosh*,* I am reaching out to you for the hundredth time*,* and you are rejecting me for the hundredth time’. (Sister*,* 28y)*

#### Support before suicide

This category includes whether the participants had any support before the suicide happened, if they experienced the support as helpful and if there were any barriers in getting help.

Overall, only two participants reported receiving professional support prior to the suicide due to their burden. One participant received support from a coach, another was seeing a psychotherapist for a short time.

*After the third session she said to me ‘you do not need any help*,* you can handle it’. (…) And then I was discharged. And I never really thought about it again. (Mother*,* 61y)*

As already mentioned in the previous category, only few caregivers talked about their stress and sought informal support. Those who did felt that support from their family, partner or social environment was helpful. Above all, the exchange about one’s own stress and the feeling of not being alone were experienced here as particularly supportive.

Several participants, however, also reported that they had not thought of getting help themselves at that moment, as they were initially only focused on supporting the person at risk. The time factor also played a role here. In addition to caring for the person at risk and their own everyday life, caregivers did not find time to seek professional help for themselves.

*When he was asleep*,* we sat down and (…) read books*,* but it was late in the evening and not during opening hours of any counseling services (…) when I think about it now*,* if we have had any appointments during a really bad phase*,* I would not have wanted to leave him alone. (Sister*,* 34y)*

#### Ideas for future support

This category summarizes ideas and suggestions for supporting informal caregivers of men in a suicidal crisis. Participants were asked to identify types of support they would have needed, the best ways to reach informal caregivers, and the key elements that should be included.

Self-help groups together with other caregivers of men at risk were mentioned most frequently. Here, the main focus lies on exchange, but also on getting the feeling of not being alone with the burden.

*Where you can be part of a group and you can see*,* how the others handle it? Just to have an idea. Just to not be alone. Not to be all alone by yourself. (Daughter*,* 45y)*

This can also reduce the feeling of loneliness. Participants mainly criticized the fact that they had looked for such groups, but they were not available. Additionally, participants emphasized the need for a clear and accessible resource of available support services for informal caregivers to facilitate easier access.

The majority of participants also named (public) education and destigmatization in society as one of the most important measures.

*So*,* it is still a taboo*,* which I find quite frightening*,* because (…) if it had all been a bit more present (…) maybe I would have been sensitized in a completely different way. (Daughter*,* 29y)*

*Definitely education. Very*,* very important. (Sister*,* 49y)*

There was a desire for both own education, but also the education of the society on suicidality in order to achieve normalization. Possibilities for education are seen via the Internet or social media, via lectures by professionals or affected individuals or also in special gatekeeper training for informal caregivers.

*And for parents or older people maybe TV-shows. It has to be more present in the media*,* so that you have to deal with this topic more. (Mother*,* 45y)*

Only two of the participants would have liked support in the context of therapy, the others saw this as a possibility but would prefer a group.

*I can only say it again and again*,* just therapy or someone where you can go to once a week*,* that would be enough. (Sister*,* 34y)*

In some cases, participants also reported that they had not needed any specific support. However, they wished that they had been more involved in their male relative’s therapy or that there had been the possibility of having therapy sessions together.

*I did not have the feeling that I would need support for myself*,* but I would have liked the clinic to ask the person ‘who are your relatives? Who is close to you?’ and that they talk with their relatives about mental health disorders and about suicide. (Sister*,* 49y)*

## Discussion

This study aimed to assess the distress of informal caregivers in suicide prevention for men focusing on their coping with their burden using the results of psychological autopsy interviews with informal caregivers bereaved by male suicide. Furthermore, the objective was to examine how caregivers articulate their experiences of stress, the types of support they utilized, and ideas for future support.

First, half of the participants reported that they were worried about their male relative before the suicide. Mainly because of their relative’s deteriorating mental health or changes in behavior. However, suicide was not perceived as a likely outcome from the majority of the participants, which reduced their level of concern. Some participants stated they were not worried due to the man’s ongoing therapy. However, informal caregivers are often not included in psychotherapy and other support services and therefore do not know whether the therapy is helpful or effective. Reasons for not including caregivers are diverse and include lack of time on part of the therapist, confidentiality or that the affected individual does not want their caregiver to be involved [1]. Only four of the participants stated that they addressed their concerns and either talked to the men at risk or sometimes to other relatives or friends. Only few talked openly about their distress mostly due to insecurities how to address their own burden as well as shame and fear of stigmatization. Those findings are in line with other studies on informal caregivers for individuals with a mental health disorder [9, 38]. A study by Azman et al. [9] showed that caregivers not only lack professional support but also informal support from their surroundings.

We also aimed at finding out how caregivers deal with the distress of the person they care for. Some participants stated that they informed themselves through website or online courses on mental health and suicidal ideation and behavior. Increased knowledge is not only associated with self-efficacy and willingness to intervene but could also increase potential prevention behavior [39, 40]. It also reduces the feeling of helplessness in caregivers. Participants also reported that they talked openly with their male relative about their mental health status and tried not pamper him but act normally. Open communication is a key element in suicide prevention giving the individual the possibility to communicate their thoughts and feelings [41, 42]. Conversely, opening up to a relative can be challenging for some individuals, as caregivers are not neutral contacts, and those affected may wish to preserve a certain self-image [7]. Participants in this study also talked about limitations in their everyday lives as a result of the mental health disorder of their male relative. One third stated that they did not leave the man alone out of fear, cancelled all appointments or worked from home. These results are in line with other studies on caregiver burden in mental health [10, 13]. A study with family caregivers in mental illnesses could show that caregivers often experience a restriction in their routine including giving up on hobbies and affecting their relationships [38].

Two participants expressed their fear about suicidal tendencies prior to the suicide. Caregivers reported experiencing significant worry for the individual, yet they did not perceive suicide as a possibility. Many expressed hope that with sufficient support, the individual’s condition would improve. Additionally, numerous caregivers found it difficult to conceive that the individual was capable of suicide, leading them to dismiss it as a plausible outcome. Another contributing factor is the differing symptomatology observed in men during suicidal crises. Many caregivers were aware of mental distress and suicidal tendencies, but the deceased male relatives often showed other symptoms that made identification difficult. While symptoms such as irritability, aggression, alcohol- and substance abuse and risk behavior are more likely to be observed in a suicidal crisis in men [43], these symptoms might not be identified as warning signs from their surroundings [44]. Informal caregivers also take on a role as gatekeepers and, through their close contact, have the opportunity to recognize changes or worsening of symptoms. However, in this study it became evident that despite numerous attempts to connect their relatives at risk with support services and caring for them, caregivers often felt helpless. Many reported that their help was rejected and that there is a lack of knowledge regarding available support options.

However, it was also particularly interesting that only women who had cared for a suicidal man responded to the call for this study. This phenomenon has already been described in many other studies [2, 45]. Women tend to take on greater responsibility when someone close to them experiences a mental health disorder, which increases their likelihood of taking on caregiving roles. A scoping review by Phillips et al. [4] further indicates that female caregivers are more prone to experiencing elevated levels of stress, shame, and burden, placing them at a higher risk of self-neglect and social isolation.

The need for support for informal caregivers in suicide prevention for men becomes apparent. To support informal caregivers of men in crisis, we developed an online program, which we evaluated as part of an RCT. This enabled us to create a program that supports and educates caregivers and provides them with strategies for crisis intervention. Participants showed a significant increase in knowledge and self-efficacy as well as a willingness to provide support [19]. We also aimed at identifying helpful support for caregivers of men received. Only two participant received professional support during that time. Other participants reported receiving support from friends, other family members or friends. Reasons for not seeking professional support were lack of time and not even thinking about the possibility of seeking help. However, it is not uncommon for caregivers to rarely seek help themselves [46, 47]. For this reason, we also tried to collect ideas for future support with this study. The wish for exchange with other caregivers in a group was prominent, which in turn would also prevent isolation and loneliness.

In addition, participants suggested providing information material on male suicidality and also campaigns against stigmatization and for educating the public. Support services for informal caregivers of people with mental health disorders are scarce, with some local support groups more commonly available in larger cities. However, in recent years there are some studies developing and evaluating support services for caregivers [46, 47]. Especially online support services are promising due to their flexibility, low-threshold accessibility, and minimal time demands meeting the needs of the caregivers.

### Strengths and limitation

One of the strengths of this study is the use of psychological autopsy interviews which is a well-proven method in suicide research and provide a detailed insight into a deceased person’s life. In addition, this is one of the few studies focusing on informal caregivers in male suicide prevention. The time criterion of 3–12 months ensures that participants can still sufficiently remember details about the time before the suicide. The sample exhibits substantial variance in both age and degree of kinship. Even if the sample of 15 participants is quite small, data saturation was achieved within this cohort. However, the sample size limits generalizability of the findings. Additionally, only women were involved in this study, and although they are increasingly taking on the role of caregiver, male caregivers do of course exist as well. Unfortunately, their views could not be reflected in this study. Despite the time criterion, some responses may be subject to recall bias or inaccuracies. The information cannot therefore be verified objectively and is shaped by the memories of the participants.

## Conclusion

Previous research has already shown that informal caregivers play a crucial role in suicide prevention but are themselves under great stress and receive little support. Our findings highlight the critical importance of contact between caregivers and men in crisis. The observed high levels of stress among caregivers can be attributed to anxiety, persistent concerns for the individual at risk, and the insufficient availability of support services. Therefore, it is imperative to urgently expand and enhance support services for informal caregivers, ensuring that this highly stressed group receives the necessary assistance.

## Data Availability

Data and material can be requested from the first author.
